# Interactions of Carvacrol, Caprylic Acid, Habituation, and Mild Heat for Pressure-Based Inactivation of O157 and Non-O157 Serogroups of Shiga Toxin-Producing *Escherichia coli* in Acidic Environment

**DOI:** 10.3390/microorganisms7050145

**Published:** 2019-05-23

**Authors:** Md Niamul Kabir, Sadiye Aras, Abimbola Allison, Jayashan Adhikari, Shahid Chowdhury, Aliyar Fouladkhah

**Affiliations:** 1 Public Health Microbiology Laboratory, Tennessee State University, Nashville, TN 37209, USA; mkabir492@gmail.com (M.N.K.); sadiyearas47@gmail.com (S.A.); abimbolaallison20@gmail.com (A.A.); adkjason99@gmail.com (J.A.); schowdh1@tnstate.edu (S.C.); 2 Cooperative Extension Program, Tennessee State University, Nashville, TN 37209, USA

**Keywords:** Shiga toxin-producing *Escherichia coli*, habituation, carvacrol, caprylic acid, high-pressure pasteurization

## Abstract

The current study investigated synergism of elevated hydrostatic pressure, habituation, mild heat, and antimicrobials for inactivation of O157 and non-O157 serogroups of Shiga toxin-producing *Escherichia coli.* Various times at a pressure intensity level of 450 MPa were investigated at 4 and 45 °C with and without carvacrol, and caprylic acid before and after three-day aerobic habituation in blueberry juice. Experiments were conducted in three biologically independent repetitions each consist of two replications and were statistically analyzed as a randomized complete block design study using ANOVA followed by Tukey- and Dunnett’s-adjusted mean separations. Under the condition of this experiment, habituation of the microbial pathogen played an influential (*p* < 0.05) role on inactivation rate of the pathogen. As an example, O157 and non-O157 serogroups were reduced (*p* < 0.05) by 1.4 and 1.6 Log CFU/mL after a 450 MPa treatment at 4 °C for seven min, respectively, before habituation. The corresponding log reductions (*p* < 0.05) after three-day aerobic habituation were: 2.6, and 3.3, respectively at 4 °C. Carvacrol and caprylic acid addition both augmented the pressure-based decontamination efficacy. As an example, *Escherichia coli* O157 were reduced (*p* < 0.05) by 2.6 and 4.2 log CFU/mL after a seven-min treatment at 450 MPa without, and with presence of 0.5% carvacrol, respectively, at 4 °C.

## 1. Introduction

The 2015–2020 dietary guidelines of the United States Department of Agriculture recommends an increase in consumption of fruits and vegetables [1]. Over the last two decades, consumption of fresh and processed produce has also been increasing [2]. Contamination of plant-based products prior to consumption is practically unavoidable due to the ubiquitous nature of microbial pathogens and complexity of producing and processing operations [3,4], leading to an array of health and economic complications such as foodborne illnesses, hospitalizations, and death episodes, as well as recalls of food products and foodborne disease outbreaks [4,5,6].

Contamination with *Escherichia coli* O157:H7 and non-O157 serogroups of Shiga toxin-producing *E. coli* are one of the leading concerns of foodborne illnesses linked with muscle- and plant-based foods [7,8,9]. In addition to the Shiga toxin-producing *E. coli* O157:H7 (STEC) that has historically been linked to an array of food recalls and outbreaks since 1990s [9], non-O157 serogroups of Shiga toxin-producing *E. coli* (nSTEC) have been gaining increasing public health significance recently due to their emergence in food chain [9,10]. The serogroups O26, O45, O103, O111, O121, and O145 (also known as the ‘Big Six’) are considered as the most epidemiologically significant foodborne serogroups of public health concern among nSTEC [11,12].

Data derived from active surveillance programs of Centers for Disease Control and Prevention [13] indicates that in the United States 3704 and 1579 laboratory confirmed cases occur annually associated with STEC and nSTEC, respectively [13]. It is further estimated that every year in the United States, STEC and nSTEC are responsible for 63,153 and 112,752 domestic foodborne infections, respectively. Among these cases, 68% of STEC and 82% of nSTEC cases are foodborne in nature [5]. From 1998 to 2017, at least 590 foodborne outbreaks in the United Sates, including 14 foodborne outbreaks in the state of Tennessee were associated with STEC and/or nSTEC [13].

Although acidification or use of acidic foods are commonly associated with limited multiplication of microorganisms [14], microbial pathogens could survive and proliferate under acidic conditions [15,16,17]. Particularly, STEC had been involved in several outbreaks of foodborne diseases in different acidic foods, for example: yoghurt [18], mayonnaise [19] and apple cider [20]. It is also observed that acid adaptation can enhance STEC ability to survive in acidic juices for example in asparagus juice (pH = 3.6) and in mango juice (pH = 3.2) [21]. As an indigenous fruit crop of North America, blueberries have particularly low pH [22], have been associated with a seven-month STEC outbreak in Massachusetts [13], and thus, could be used as a model for investigating validation studies against STEC and nSTEC in acidic environment.

A viable alternative for pasteurization of products in manufacturing is application of elevated hydrostatic pressure [23]. Unlike traditional thermal processing methods that are typically associated with undesirable physiochemical and organoleptic changes in treated products [24], pressure-based pasteurization could be utilized for assuring safety of the products while minimally affecting their sensory and nutritional composition [25,26]. A pressure-based pasteurization could utilizes hydrostatic pressure of 100 to 1000 MPa, pressure-intensity level of around 600 MPa (87 K PSI) for about three min are currently the most common treatment in the private industry [27]. The main challenge for further adaption of pressure-based pasteurization treatments is slightly higher processing costs associated with the technology, thus, application of pressure treatments at intensity levels below 600 MPa, augmented with mild heat and natural antimicrobials could be a desirable approach for the food industry [27].

Caprylic acid is an eight-carbon fatty acid, which could be naturally found in several foods (coconut oil, bovine milk, palm oil, etc.) and is *Generally Recognized as Safe* by the U.S., Food and Drug Administration as a food additive [28,29]. Caprylic acid (C_8_H_16_O_2_) could be an effective antimicrobial compound against Gram-negative and Gram-positive foodborne pathogens such as *E. coli* O157: H7, *Listeria monocytogenes* and *Salmonella* serovars [28,30,31,32]. Carvacrol (C_10_H_14_O), found primarily in oregano, is another natural bioactive compound with reported antimicrobial properties [33] and is broadly known for its effective antioxidant and antimicrobial activity [34,35].

The purpose of this study was to investigate the role of mild heat and addition of caprylic acid and carvacrol on decontamination efficacy of a pressure-based pasteurization treatment against STEC and nSTEC. Habituation of the pathogen, as further delineated in Section 2.1, in an acidic food vehicle were also investigated as an important element for maximizing external validity of a decontamination hurdle validation study.

## 2. Materials and Methods

### 2.1. Escherichia coli Strains, Preparation of Culture, Habituation, and Inoculation

A six-strain mixture of Shiga toxin-producing *E. coli* O157:H7 (STEC) (ATCC^®^, Manassas, VA, USA, numbers BAA 460, 43888, 43894, 35150, 43889 and 43890) and a six-strain mixture of ‘Big Six’ non-O157 Shiga toxin-producing *E. coli* (nSTEC) strains, including O26:H11, O45:H2, O103:H2, O111:NM, O121:H19, and O145 (ATCC^®^ numbers BAA 2196, BAA 2193, BAA 2215, BAA 2440, BAA 2219 and BAA 2192 respectively) were used in this study for inoculation of sterilized (autoclaved at 121 °C, for 15 min, under 15 PSI) blueberry juice. The STEC and nSTEC strains with public health significance and those derived from our previously published strain selection trials were selected for this study [9].

The cultures for each of the above-mentioned strains, obtained from American Type Culture Collection (Manassas, VA, USA), were grown on Tryptic Soy Agar (Difco, Becton Dickinson, Franklin Lakes, NJ, USA) supplemented with 0.6% yeast extract (TSA + YE) and for 24 h incubated at 37 °C. Forty eight hours before each experiment, a loopful of single colony of each STEC or nSTEC strains was aseptically transferred for activation into 10 mL Tryptic Soy Broth (Difco, Becton Dickinson, Franklin Lakes, NJ, USA) supplemented with 0.6% yeast extract (TSB + YE). Use of this media and the supplement minimizes acid stress of the bacterial cells during incubation at 37 °C for 20–24 h [23,27,36]. After incubation for 20–24 h at 37 °C, 100-µL aliquot of the culture was individually and aseptically sub-cultured into another 10 mL of TSB + YE, for 22–24 h at 37 °C, for each of the 12 strains, separately.

Each overnight sub-cultured strain (2 mL per strain) was then harvested by centrifugation (Model 5424, Eppendorf North America, Hauppauge, NY, USA; Rotor FA-45-24-11) at 6000 RPM (3548 *g* for 88 mm rotor) for 15 min. Bacterial pellets were then re-suspended in 2 mL Phosphate Buffered Saline (VWR International, Radnor, PA, USA) and washed twice by centrifugation with the above-mentioned intensity and time to remove growth media, excreted secondary metabolites, and sloughed cell components. Two separate six-strain bacterial cocktails (for STEC and nSTEC) were made by combining the washed and re-suspended strains into PBS (VWR International, Radnor, PA, USA), and were used as the inocula for this study. Non-habituated samples were prepared by 10-fold dilution of each of the STEC and nSTEC cocktails in PBS followed by inoculating sterilized blueberry juice samples for target population of 5–6 Log CFU/mL. The habituated samples were prepared by adding 10 mL of STEC and nSTEC cocktails (separately for each strain mixture) to 40 mL of sterilized blueberry juice, followed by a 72 h aerobic storage at 4 °C [23]. Habituation allows pathogen acclimatization to intrinsic factor and temperature of the food product and could impact external validity of a microbial challenge study [37,38,39]. Levels of inoculation for habituated and non-habituated samples and below-mentioned temperatures and concentrations of antimicrobials were selected after conduct of preliminary trials.

### 2.2. Preparation of Antimicrobials, and Mild Heat and Pressure-Based Pasteurization

Two naturally occurring antimicrobial compounds (carvacrol and caprylic acid) were used in this study for inactivation of 72-h habituated STEC and nSTEC in sterilized blueberry juice at two temperatures and at an elevated hydrostatic pressure level of 450 MPa. The temperature of the trials were precisely controlled using a water jacket surrounding the treatment chamber, connected to a circulating water bath and monitored by k-type thermocouples as delineated in details in our recent open access publications [23,27]. For 4 °C experiments, 0.5% (7.5 µL of antimicrobial in 1.5 mL of inoculated product (*v*/*v*)) and for 45 °C experiment, 0.1% concentration (1.5 µL of antimicrobial in 1.5 mL of inoculated product (*v*/*v*)) of carvacrol and caprylic acid were used based on the above-mentioned preliminary trials. In each experiment, the concentration of antimicrobials was prepared aseptically in sterilized blueberry juice. Inoculated blueberry juice were then exposed to 450 Megapascal (MPa), i.e., c. 65,000 pounds per square inch (PSI) hydrostatic pressure (Barocycler Hub880 Explorer, Pressure Bioscience Inc., South Easton, MA, USA) at 4 and 45 °C for the time intervals of 0 (untreated control) to 7 min. Samples containing antimicrobials were also tested immediately after addition of the antimicrobial and prior to pressure treatment (treated control). The treatments were carried out in no-disk PULSE (Pressure BioScience Inc., South Easton, MA, USA) containing 1.5 mL of inoculated blueberry juice. The PULSE tubes were then used for hydrostatic pressure treatment with 1, 3, 5 and 7 min holding time, in addition to the above-mentioned controls. Pressure and temperature of trials were monitored and recorded automatically every 3 s using HUB Explorer PBI (Version 1.0.8, Pressure BioScience Inc., South Easton, MA, USA) software.

### 2.3. The pH, Neutralization, and Microbiological Analyses

Each treated sample was neutralized using 5 mL of D/E neutralizing broth (Difco, Becton Dickinson, Franklin Lakes, NJ, USA) to reduce the effect of food vehicle’s intrinsic factors before microbiological analyses. The detection limit of microbiological analyses was, thus, 0.48 log CFU/mL. After neutralization, to enhance the recovery of injured cells, samples were 10-fold serially diluted in Maximum Recovery Diluent (Difco, Becton Dickinson, Franklin Lakes, NJ, USA) and then plated on TSA media supplemented with 0.6% yeast extract (TSA + YE). All plates were incubated for 24–48 h at 37 °C. After incubation, colony forming units were counted manually and converted into log values for further statistical analyses. The pH of treated samples was measured two times (after treatment and before neutralization, as well as after neutralization) using a digital pH meter (Mettler Toledo AG, Grelfensee, Switzerland) calibrated at pH levels of 4, 7 and 10 before measurements.

### 2.4. Statistical Analyses and Experimental Design

The sample size of this study was determined to be at least 5 repetitions per treatment to achieve statistical power of 80%. This sample size was obtained from a previous *a priori* power analysis using Proc Power of SAS software (version 9.2, SAS Institute, Cary, NC, USA) using existing pressure-treated products in the public health microbiology laboratory [40]. The present study was conducted at two temperatures of 4 and 45 °C using two inocula of STEC and nSTEC. At each temperature, the study contained three biologically independent repetitions (three blocks), each consisted of 2 replications. Each replication was also microbiologically analyzed in duplicate (microbiological replications). Thus each reported value is a mean of 12 individual analyses (i.e., 3 blocks, 2 replications, and 2 microbiological repetitions). Initial data arrangement, log transformations and descriptive analysis of the data were completed using Microsoft Excel. The study was considered as a randomized complete block design, and log-transformed microbial counts were statistically analyzed using generalized liner model of SAS for conduct of ANOVA followed by Tukey- and Dunnett’s-adjusted mean separations at type I error level of 5% (alpha= 0.05). In order to calculate inactivation indices (D-value and K_max_) Microsoft Excel and GInaFiT (version 1.7, Katholieke Universiteit, Leuven, Belgium) [41] software were used, respectively.

## 3. Results and Discussion

As previously delineated in Section 2.2, the experiments were conducted under controlled temperatures to assure microbial inactivation could be attributed to the intrinsic and extrinsic factors of interest rather than temperature fluctuations. Samples treated at 4 and 45 °C, had similar (*p* ≥ 0.05) temperature values (mean ± SD) before and after the treatments. Across all treatments at 4 °C, the values before treatments were 4.8 ± 0.2 °C and were 4.9 ± 0.2 °C after the treatments. Values were ranging from 4.3 to 5.2 °C and 4.3 to 5.3 °C, before and after treatments, respectively. For samples treated at 45 °C as well, temperature recordings were similar (*p* < 0.05) before and after treatments. The temperature values were 44.5 ± 0.3 and 44.8 ± 0.4 °C, before and after treatments, respectively. The range for the recordings were from 43.7 to 45.0 °C and 43.7 to 45.2 °C for samples prior and after treatments, respectively. Extent of precision in control of temperature could be further delineated through calculation of coefficient of variation (CV) associated with the temperature recordings. The CVs associated with 4 °C samples were 4.51% and 4.57% and for samples treated at 45 °C were 0.58% and 0.76%, before and after treatments, respectively.

The pH levels of the samples were also similar (*p* ≥ 0.05) before and after treatments. For samples treated at 4 °C, and prior to neutralization, the pH value (mean ± SD) and range were 3.16 ± 0.0 and 3.12 to 3.22, respectively. After neutralization, these values were expectedly increased (*p* < 0.05) to 5.56 ± 0.27, ranging from 5.25 to 6.02. Similarly, for samples treated at 45 °C, these values were 3.33 ± 0.1 and 5.54 ± 0.1, before and after neutralization. These values were ranging from 3.24 to 3.44 and 5.37 to 5.69 before and after neutralization, respectively. The CVs associated with pH measurements were 0.42% (4 °C samples, without neutralization), 4.89% (neutralized 4 °C samples), 1.61% (45 °C samples, without neutralization), and 1.70% (neutralized, 45 °C samples).

### 3.1. Pressure-Based Pasteurization of O157 and Non-O157 Serogroups of Shiga Toxin-Producing Escherichia coli at 4 °C, Before and After Habituation

**Figure 1:**
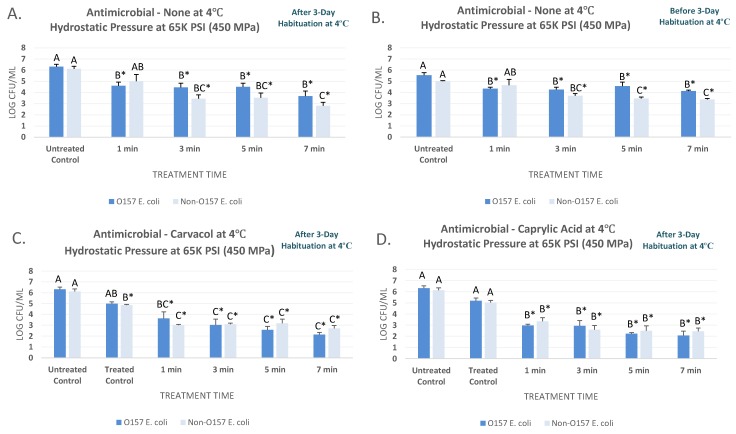
Inactivation of six-strain cocktail of habituated and non-habituated *E. coli* O157:H7 (ATCC^®^ numbers BAA 460, 43888, 43894, 35150, 43889, 43890) and the ‘Big Six’ non-O157 *E. coli* mixtures (ATCC^®^ numbers BAA 2196, BAA 2193, BAA 2215, BAA 2440, BAA 2219, BAA 2192) in sterilized blueberry juice, treated by carvacrol (0.5%), caprylic acid (0.5%) and elevated hydrostatic pressure at 450 MPa (Barocycler Hub880 Explorer, Pressure Bioscience Inc., South Easton, MA, USA) for 0, 1, 3, 5, and 7 min at 4 °C. In each graph, and for each pathogen mixture separately, columns of each time interval followed by different uppercase letters are representing log CFU/mL values (Mean ± SE) that are statistically (*p* < 0.05) different (Tukey-adjusted ANOVA). Uppercase letters followed by * sign are statistically (*p* < 0.05) different than the untreated control (not treated with antimicrobial) (Dunnett’s-adjusted ANOVA). (**A**) After 3 days of habituation, treated by no antimicrobial at 4 °C; (**B**) Before 3 days of habituation, treated by no antimicrobial at 4 °C; (**C**) After 3 days of habituation, treated by 0.5% carvacrol at 4 °C; (**D**) After 3 days of habituation, treated by 0.5% caprylic acid at 4 °C.

As further delineated in Section 2.1, this study utilized two separate inoculated products for the pressure-based microbial challenge studies using a six-strain mixture of O157 Shiga toxin-producing *Escherichia coli* (STEC) and a six-strain non-O157 mixture of O26, O45, O103, O111, O121, and O145 Shiga toxin-producing *Escherichia coli* (nSTEC). Data associated with the current study is also provided as a supplementary file. At 4 °C and after the habituation, the STEC and nSTEC counts (mean ± SD) of blueberry juice were 6.32 ± 0.5 and 6.12 ± 0.6 Log CFU/mL, respectively (Figure 1A). Hydrostatic pressure treatment of 450 MPa (c. 65 K PSI), for 1, 3, 5, and 7 min, reduced the STEC by 1.7 to 2.6 log CFU/mL and specifically reduced (*p* < 0.05) the STEC counts to 4.60 ± 0.8, 4.45 ± 0.9, 4.51 ± 0.8, 3.68 ± 1.1, respectively (Figure 1A). Sensitivity of nSTEC were similar to STEC- the treatments for 1, 3, 5, and 7 min at the above-referenced pressure and intensity level lead to 1.1, 2.7, 2.6, and 3.3 log reductions of nSTEC samples (Figure 1A). Under the condition of our experiment, habituation played an influential role on sensitivity of both STEC and nSTEC serogroups to pressure-based treatments at 4 °C (Figure 1A,B). For non-habituated samples at 4 °C, STEC and nSTEC counts were 5.55 ± 0.6 and 5.00 ± 0.1 prior to treatments, respectively. The STEC were reduced (*p* < 0.05) to 4.35 ± 0.3, 4.26 ± 0.5, 4.57 ± 0.9, and 4.13 ± 0.2 log CFU/mL, after treatments for 1, 3, 5, and 7 min at 450 MPa, respectively (Figure 1B). These reductions were considerably less that reductions of the habituated STEC. In other words, the habituated STEC were more sensitive to pressure-based treatments at this temperature relative to the non-habituated phenotype. As an example, 7 min of treatment at 450 MPa at 4 °C reduced the habituation STEC (*p* < 0.05) by 3.7 log CFU/mL (Figure 1A), while the same treatment were only capable of reducing (*p* < 0.05) the non-habituated STEC for 1.4 log CFU/mL (Figure 1B). This trend was also observed for habituated and non-habituated nSTEC (Figure 1A,B).

This considerable difference in sensitivity of the pathogen before and after habituation had been discussed in the microbiology literature in the past. While studies, similar to our current study, had observed that post-stress, pathogens exhibit more sensitivity to a decontamination treatment. Some studies also indicate certain stressors could lead to cross-protective effects, i.e., increasing the tolerance of a pathogen post-stress [23,38,42,43]. If a manufacturer is relying on validation studies with non-habituated inoculated pathogen, the validation data could be an overestimation or underestimation of the treatment decontamination efficacy, and thus, leading to false sense of treatment efficacy or a treatment that is overly conservative. This could also lead to over- or under-estimation of microbial reductions in risk assessment analyses throughout the supply chain. It is thus recommended that habituation for each specific product-pathogen-treatment combination be considered as an important factor of a validation study to assure data obtained from a microbial challenge study has external validity and is conducted in an environment that is as close as possible to actual processing condition of a product. This could assure economic feasibility of a treatment as well as providing assurance that a treatment is safeguarding the public health. Currently, there is a knowledge gap about sensitivity of acid-adapted and acid-stressed foodborne pathogens of public health concern to various pressure-based treatments relative to their wild-type phenotypes.

### 3.2. Augmenting the Efficacy of High Pressure Pasteurization using Carvacrol and Caprylic Acid at 4 °C

Under the condition of our experiments, we observed the selected two natural antimicrobials could appreciably augment the efficacy of the pressure-based pasteurization of STEC and nSTEC at 4 °C. It is noteworthy that the synergism of elevated hydrostatic pressure and carvacrol and caprylic acid were investigated on inoculated samples with three-day aerobic habituation that, as discussed in Section 3.1, yields more realistic outcome with higher external validity. Data and graphical representations obtained and reported for these experiments were similar in structure to those elaborated in Section 3.1 with the exception that the microbial reductions immediately after exposure to 0.5% antimicrobial were also determined, thus graphs contain untreated controls as well as treated controls (e.g., samples that are immediately neutralized and enumerated after exposure to the antimicrobial).

The STEC and nSTEC counts (mean ± SD) for untreated controls were 6.32 ± 0.5 and 6.12 ± 0.6 log CFU/mL, respectively at 4 °C. Immediately after exposure to 0.5% carvacrol, these counts were reduced (*p* <0.05) to 4.99 ± 0.4 and 4.86 ± 0.1 log CFU/mL, for STEC and nSTEC samples, respectively (Figure 1C). Carvacrol were able to enhance (*p* < 0.05) the efficacy of the treatment. As an example, treatments of STEC samples for 5 and 7 min at 450 MPa at 4 °C lead to 3.8 and 4.2 log CFU/mL reductions (*p* < 0.05) while same treatment at the same temperature and intensity level without presence of carvacrol resulted in 1.0 and 1.4 log CFU/mL reductions (*p* < 0.05) in habituated samples, respectively (Figure 1A,C). In vast majority of tested time intervals, STEC and nSTEC serogroups exhibited comparable sensitivity to high hydrostatic pressure (Figure 1A–D). Caprylic acid, at 0.5% concentration, were similarly effective to augment the decontamination efficacy of the pressure-based treatments at 4 °C. The nSTEC counts, as an example, were 6.12 ± 0.6 log CFU/mL prior to treatment and prior to exposure to caprylic acid (untreated control). These counts were reduced (*p* < 0.05) to 5.02 ± 0.5 log CFU/mL immediately after exposure to 0.5% caprylic acid (treated controls) and were further reduced (*p* < 0.05) to 3.35 ± 0.8, 2.60 ± 0.9, 2.49 ± 1.1, 2.44 ± 0.8 log CFU/mL after 1-, 3-, 5-, and 7-min treatments at 450 MPa at 4 °C (Figure 1D). These reductions were appreciably higher than those obtained from elevated hydrostatic pressure alone for both STEC and nSTEC. As an example, the above-reference 7-min treatment reduced (*p* < 0.05) the STEC and nSTEC for 4.2 and 3.7 log CFU/mL in presence of 0.5% caprylic acid, respectively, while the same treatment resulted in 1.4 and 1.6 log CFU/mL reductions (*p* < 0.05) for the habituated samples without caprylic acid (Figure 1A,D).

These results could be of practical importance for the private industry with a high-pressure processing plant. At current times, slightly higher operation costs of many pressure-treated products relative to existing heat-treated commodities in the market are the main curtailment for further expanding the utilization of this technology in the food processing industry [23,27]. Main costs of the operation are associated with maintenance and energy expenditure associated with use of high levels of hydrostatic pressure. Our study indicates that lower levels of pressure could lead to similar decontamination efficacy in presence of natural antimicrobials such as carvacrol and caprylic acid.

### 3.3. Pressure-Based Pasteurization of the Pathogen at 45 °C as Affected by Habituation, Carvacrol and Caprylic Acid

**Figure 2:**
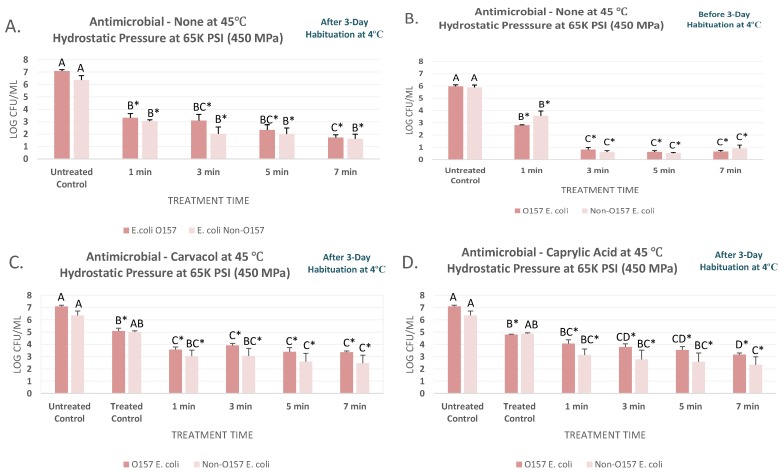
Inactivation of six-strain cocktail of habituated and non-habituated *E. coli* O157:H7 (ATCC^®^ numbers BAA 460, 43888, 43894, 35150, 43889, 43890) and the ‘Big Six’ non-O157 *E. coli* strain mixtures (ATCC^®^ numbers BAA 2196, BAA 2193, BAA 2215, BAA 2440, BAA 2219, BAA 2192) in sterilized blueberry juice, treated by carvacrol (0.1%), caprylic acid (0.1%) and elevated hydrostatic pressure at 450 MPa (Barocycler Hub880 Explorer, Pressure Bioscience Inc., South Easton, MA, USA) for 0, 1, 3, 5, and 7 min at 45 °C. In each graph, and for each pathogen mixture separately, columns of each time interval followed by different uppercase letters are representing log CFU/mL values (mean ± SE) that are statistically (*p* < 0.05) different (Tukey-adjusted ANOVA). Uppercase letters followed by * sign are statistically (*p* < 0.05) different than the untreated control (not treated with antimicrobial) (Dunnett’s-adjusted ANOVA). (**A**) After three days of habituation, treated by no antimicrobial at 45 °C; (**B**) Before three days of habituation, treated by no antimicrobial at 45 °C; (**C**) After three days of habituation, treated by 0.1% carvacrol at 45 °C; (**D**) After three days of habituation, treated by 0.1% caprylic acid at 45 °C.

The pressure treatments discussed in Section 3.1 and Section 3.2, coupled with mild heat were appreciably more efficacious for decontamination of the product from STEC and nSTEC (Figure 2A–C). This thermal-assisted pressure-based treatment at 450 MPa and 45 °C were able to reduce (*p* < 0.05) the STEC counts by 3.8, 4.0, 4.8, and 5.4 log CFU/mL for habituated samples (Figure 2A). This decontamination efficacy were also observed with similar trends for the nSTEC samples, leading to 3.3 to 4.8 log CFU/mL reductions for treatments of up to 7 min (Figure 2A). Effects of habituation at this temperature were less pronounced relative to the experiment conducted at 4 °C (Figure 2A). As an example, counts of non-habituated STEC and nSTEC samples were 5.96 ± 0.3 and 5.88 ± 0.5 before treatments and were reduced (*p* < 0.05) to 0.66 ± 0.2 and 0.91 ± 0.7 log CFU/mL after 7-min treatments at 450 MPa and 45 °C, respectively. Counts for habituated STEC and nSTEC were reduced (*p* < 0.05) by 5.4 and 4.8 log values, similar to the reductions obtained by treatment of non-habituated samples (Figure 2A,B). Our data indicates, habituation could have a more pronounced effect on external validity of a pressure-based validation study at 4 °C while may have only modest effects on validity of a thermal-assisted high-pressure processing.

At elevated temperature, effects of carvacrol and caprylic acid at 0.1% were also less pronounced in augmenting the decontamination efficacy of the treatments (Figure 2C,D). This indicates that while these antimicrobials might be efficacious alone, or coupled with pressure-based treatments at lower temperature, at 0.5% concentrations, but these do not augment the efficacy of a treatment at higher temperature when tested at 0.1%. Similar effects were observed in the past when acidic acid was not able to augment efficacy of a heat treatment at elevated temperature while efficacious at ambient environment [44]. As an example, STEC counts of habituated samples treated without antimicrobial, with 0.1% carvacrol, and with 0.1% caprylic acid for 3 min at 450 MPa were similar (*p* ≥ 0.05) and were 3.09 ± 1.3, 3.91 ± 0.4, and 3.79 ± 0.6 log CFU/mL, respectively (Figure 2A,C,D). Similar to treatments at lower temperature, STEC and nSTEC counts were comparable for the vast majority of time and pressure treatments, prior and after habituation, and in presence or absence of the antimicrobials (Figure 2A–D). Our results, thus indicate that mild elevated heat and natural antimicrobial could augment efficacy of a pressure-based pasteurization with similar effectiveness against STEC and nSTEC, but utilization of both mild heat and antimicrobials simultaneously does not necessarily provide added decontamination benefit.

### 3.4. Linear and Non-Leaner Inactivation Indices for High Pressure Pasteurization of O157 and Non-O157 Serogroups of Shiga Toxin-Producing Escherichia coli at 4 and 45 °C

**Figure 3:**
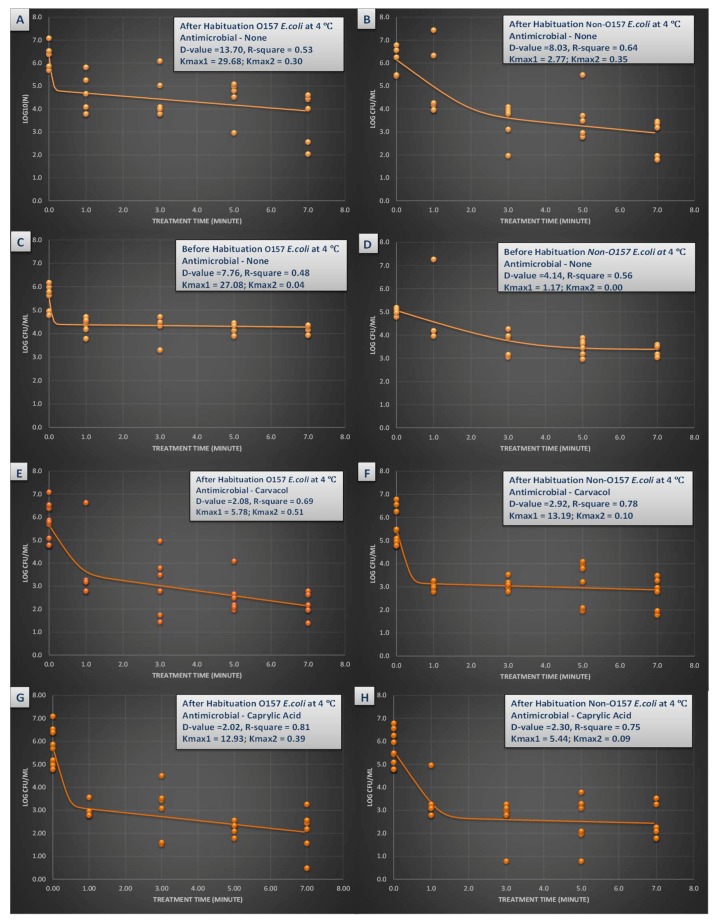
Inactivation rates for six-strain habituated and non-habituated mixture of *E. coli* O157:H7 (ATCC^®^ numbers BAA 460, 43888, 43894, 35150, 43889, 43890) and the ‘Big Six’ non-O157 *E. coli* strain mixtures (ATCC^®^ numbers BAA 2196, BAA 2193, BAA 2215, BAA 2440, BAA 2219, BAA 2192) exposed to 0.5% carvacrol, 0.5% caprylic acid, and elevated hydrostatic pressure at 450 MPa (Barocycler Hub 440, Pressure BioScience Inc., South Easton, MA) in sterilized blueberry juice at 4 °C. Using the GInaFiT software, the provided K_max_ values are selected from the best-fitted model (goodness-of-fit indicator of R^2^ values, α = 0.05). K_max_ values indicate the expressions of number of log cycles of reduction in 1/min unit for each pressure/temperature combinations. Presented D-values are calculated based on best-fitted linear model, showing time required for one log (90%) of microbial cell reductions of the microbial cell mixture. (**A**) Habituated *E. coli* O157 treated by no antimicrobial at 4 °C with R^2^ = 0.53; (**B**) Habituated *E. coli* non-O157 treated by no antimicrobial at 4 °C with R^2^ = 0.64; (**C**) Non-habituated *E. coli* O157 treated by no antimicrobial at 4 °C with R^2^ = 0.48; (**D**) Non-habituated *E. coli* non-O157 treated by no antimicrobial at 4 °C with R^2^ = 0.56; (**E**) Habituated *E. coli* O157 treated by carvacrol (0.5%) at 4 °C with R^2^ = 0.69; (**F**) Habituated *E. coli* non-O157 treated by carvacrol (0.5%) at 4 °C with R^2^ = 0.78; (**G**). Habituated *E. coli* O157 treated by caprylic acid (0.5%) at 4 °C with R^2^ = 0.81; (**H**). Habituated *E. coli* non-O157 treated by caprylic acid (0.5%) at 4 °C with R^2^ = 0.75.

**Figure 4:**
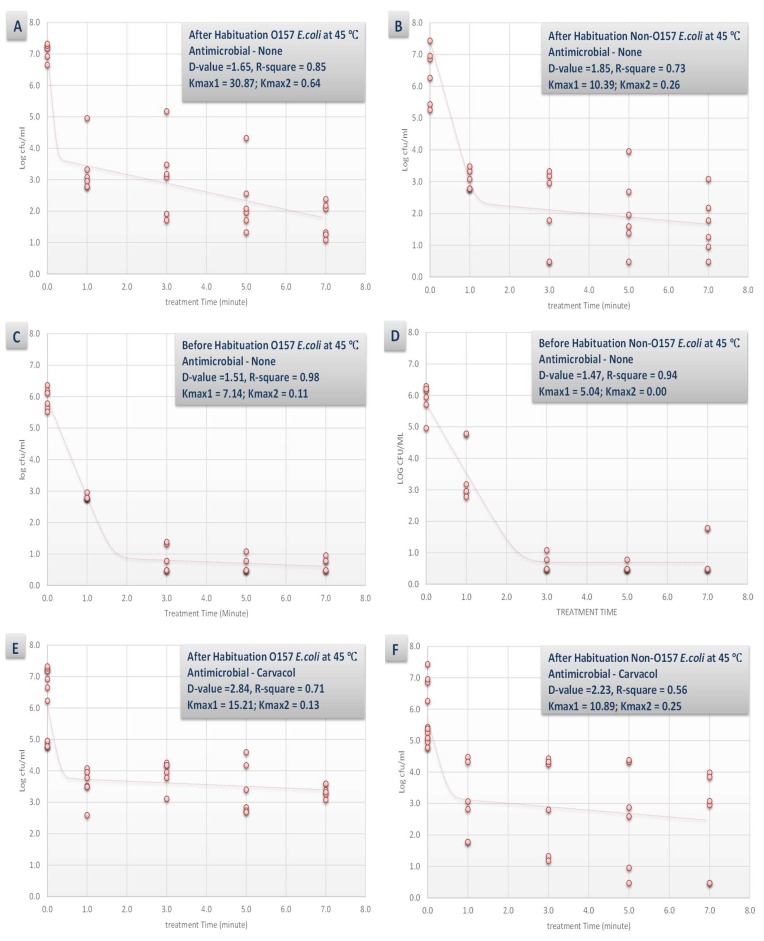

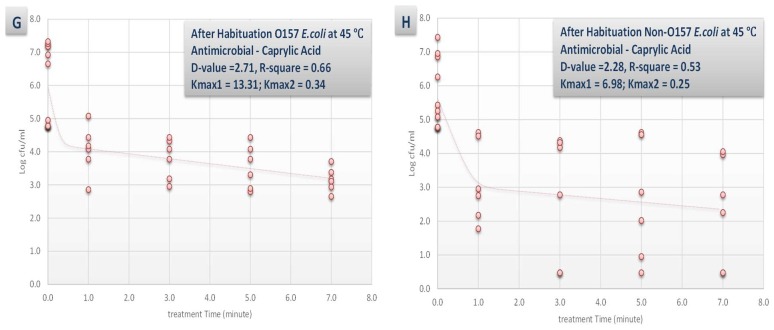
Inactivation rates for six-strain habituated and non-habituated mixture of *E. coli* O157:H7 (ATCC^®^ numbers BAA 460, 43888, 43894, 35150, 43889, 43890) and the ‘Big Six’ non-O157 *E. coli* strain mixtures (ATCC^®^ numbers BAA 2196, BAA 2193, BAA 2215, BAA 2440, BAA 2219, BAA 2192) exposed to 0.1% carvacrol, 0.1% caprylic acid, and elevated hydrostatic pressure at 450 MPa (Barocycler Hub 440, Pressure BioScience Inc., South Easton, MA) in sterilized blueberry juice. Using the GInaFiT software, the provided K_max_ values are selected from the best-fitted model (goodness-of-fit indicator of R^2^ values, α = 0.05). K_max_ values indicate the expressions of number of log cycles of reduction in 1/min unit for each pressure/temperature combinations. Presented D-values are calculated based on best-fitted linear model, showing time required for one log (90%) of microbial cell reductions of the habituated microbial cell mixture. (**A**) Habituated *E. coli* O157 treated by no antimicrobial at 45 °C with R^2^ = 0.53; (**B**) Habituated *E. coli* non-O157 treated by no antimicrobial at 45 °C with R^2^ = 0.64; (**C**) Non-habituated *E. coli* O157 treated by no antimicrobial at 45 °C with R^2^ = 0.48; (**D**) Non-habituated *E. coli* non-O157 treated by no antimicrobial at 45 °C with R^2^ = 0.56; (**E**) Habituated *E. coli* O157 treated by carvacrol (0.1%) at 45 °C with R^2^ = 0.69; (**F**) Habituated *E. coli* non-O157 treated by carvacrol (0.1%) at 45 °C with R^2^ = 0.78; (**G**) Habituated *E. coli* O157 treated by caprylic acid (0.1%) at 45 °C with R^2^ = 0.81; (**H**) Habituated *E. coli* non-O157 treated by caprylic acid (0.1%) at 45 °C with R^2^ = 0.75.

Effects of habituation and synergism of heat, carvacrol and/or caprylic acid with the pressure-based pasteurization could be further discussed by interpretation of linear and non-linear inactivation indices (Figure 3 and Figure 4). D-value was the linear model utilized in this study that could be interpreted as the time required at the specific condition of the experiment to achieve 90% reduction of the inoculated pathogen (i.e., one-log reduction). A non-linear model had also been utilized in this study using GlnaFiT version 1.7 software [41]. The reported k_max_ values are in unit of 1/min thus smaller K_max_ values indicate longer time required for reduction of the pathogen, in contrast to D-value that is in unit of min.

The D-value for STEC for habituated and non-habituated samples (Figure 3A,C) emphasizes on importance of this practice on outcome of a challenge study. The D-value associated with habituated STEC were 13.70 min while for non-habituated samples this inactivation index was 7.76 min (Figure 3A,C). This effect was not observed at higher temperature. At 45 °C, the D-values were similar for habituated and non-habituated STEC samples and were 1.65 and 1.51 min, respectively (Figure 3A,C).

Carvacrol was able to augment the efficacy of pressure-based pasteurization of the pathogen as evidenced by inactivation indices. As an example, nSTEC required 8.03 min of treatment at 450 MPa and 4 °C for one-log reduction e.g., D-value = 8.03 min (Figure 3B). In presence of 0.5% carvacrol, same treatment required only 2.92 min for one-log reduction (Figure 3F). The k_max_ values also delivered similar trend, having values of 2.77 and 13.19 1/min for nSTEC samples without carvacrol, and those treated with presence of 0.5% carvacrol (Figure 3B,F). At 4 °C, 0.5% caprylic acid was also capable of reducing the time for one-log reduction of both STEC and nSTEC as exhibited in Figure 3A,B,G,H.

The synergistic effects of the tested antimicrobial (0.1% concentration) and habituation were less pronounced at elevated temperature of 45 °C (Figure 4). For example, the D-values for habituated with no antimicrobial, non-habituated with no antimicrobial, habituated and treated with 0.1% carvacrol, and habituated and treated with 0.1% caprylic acid for STEC samples were similar (*p* < 0.05) and were 1.65, 1.51, 2.84, and 2.71 min, respectively (Figure 4A,C,E,G). This indicates that additional of antimicrobials could appreciable enhance the decontamination efficacy of a pressure-based intervention at 4 °C while could have minor to no effects for augmenting the efficacy of a thermal-assisted high pressure pasteurization.

As further discussed in the introduction, antimicrobials used in the current study have *Generally Recognized as Safe* status in the United States regulatory landscape [28,45] and the concentrations utilized are similar to those used previously in literature [46]. As for any product development project, incorporation of these antimicrobials in a product formula for enhancing safety of the product, requires product specific and close attention to organoleptic properties of the product with and without the antimicrobials.

It is also noteworthy that this study utilized a six-strain mixture of *E. coli* O157:H7 and a six-strain mixture of non-O157 Shiga toxin-producing *E. coli*. As delineated in Section 2.1, these were selected based on our previously published screening trials as well as the strains’ public health significance. Acid tolerance, sensitivity to intrinsic and extrinsic factors of a product, and reduction as a result of a thermal or non-thermal treatment could vary immensely among the plethora of Shiga toxin-producing isolates of the pathogen. Conducting experiments with similar design to the current study in future, using an array of individual strains followed by further analyses of the survivors after the treatments could be experiments of utmost importance and a complement to the current study for better assimilation of sensitivity of this pathogen of public health concern to pressure-based interventions under various intrinsic and extrinsic conditions of a product and processing conditions.

## 4. Conclusions

Under the condition of our experiments, for the vast majority of tested time and pressure intervals in presence or absence of two antimicrobials, O157 and non-O157 serogroups of Shiga toxin-producing *Escherichia coli* exhibited similar sensitivity to elevated hydrostatic pressure. Thus, if a pressure-based treatment is validated and is efficacious for decontamination of O157 serogroups of *Escherichia coli*, it would almost certainly exhibit comparable efficacy for reduction of non-O157 serogroups of the pathogen as well. We also observed that, particularly for treatments at 4 °C, habituation of samples could meaningfully alter the results of a microbial challenge study and thus would need to be carefully considered for maximizing the external validity of a validation study. Reducing the cost of pressure-based treatments are currently the major curtailment for further adaption of this emerging technology. Our study indicates that application of natural antimicrobials could augment the decontamination efficacy of this technology, allowing the practitioners to benefit from synergism of natural antimicrobials and elevated hydrostatic pressure, to utilize lower intensity of the treatment with the same level of microbiological safety. This could be a practical solution for ultimately reducing high-pressure processing operation costs and increasing the competitiveness of products manufactured with this technology. This could also lead to enhanced preservation of nutritional and sensory properties of the products since mild hydrostatic pressure treatments are typically associated with no or minimal deleterious effects on physiochemical and organoleptic properties of food products.

## Supplementary Materials

Supplementary materials can be found at https://www.mdpi.com/2076-2607/7/5/145/s1.
